# A 30-Year Prospective Follow-Up Study Reveals Risk Factors for Early Death in Cartilage-Hair Hypoplasia

**DOI:** 10.3389/fimmu.2019.01581

**Published:** 2019-07-16

**Authors:** Svetlana Vakkilainen, Mervi Taskinen, Paula Klemetti, Eero Pukkala, Outi Mäkitie

**Affiliations:** ^1^Pediatric Research Center, Children's Hospital, University of Helsinki and HUS Helsinki University Hospital, Helsinki, Finland; ^2^Folkhälsan Research Center, Institute of Genetics, Helsinki, Finland; ^3^Research Program for Clinical and Molecular Metabolism, Faculty of Medicine, University of Helsinki, Helsinki, Finland; ^4^Faculty of Social Sciences, Tampere University, Tampere, Finland; ^5^Department of Molecular Medicine and Surgery, Karolinska Institutet, Clinical Genetics, Karolinska University Hospital, Stockholm, Sweden; ^6^Department of Clinical Genetics, HUSLAB, Helsinki University Hospital, Helsinki, Finland

**Keywords:** cancer, combined immunodeficiency, adult-onset immunodeficiency, lung disease, lymphoma, mortality, RMRP, skeletal dysplasia

## Abstract

Cartilage-hair hypoplasia (CHH) is a skeletal dysplasia with combined immunodeficiency, variable clinical course and increased risk of malignancy. Management of CHH is complicated by a paucity of long-term follow-up data, as well as knowledge on prognostic factors. We assessed clinical course and risk factors for mortality in a prospective cohort study of 80 patients with CHH recruited in 1985–1991 and followed up until 2016. For all patients we collected additional health information from health records and from the national Medical Databases and Cause-of-death Registry. The primary outcome was immunodeficiency-related death, including death from infections, lung disease and malignancy. Standardized mortality ratios (SMRs) were calculated using national mortality rates as reference. Half of the patients (57%, *n* = 46) manifested no symptoms of immunodeficiency during follow-up while 19% (*n* = 15) and 24% (*n* = 19) demonstrated symptoms of humoral or combined immunodeficiency, including six cases of adult-onset immunodeficiency. In a significant proportion of patients (17/79, 22%), clinical features of immunodeficiency progressed over time. Of the 15 patients with non-skin cancer, eight had no preceding clinical symptoms of immunodeficiency. Altogether 20 patients had deceased (SMR = 7.0, 95%CI = 4.3–11); most commonly from malignancy (*n* = 7, SMR = 10, 95%CI = 4.1–21) and lung disease (*n* = 4, SMR = 46, 95%CI = 9.5–130). Mortality associated with birth length below −4 standard deviation (compared to normal, SMR/SMR ratio = 5.4, 95%CI = 1.5–20), symptoms of combined immunodeficiency (compared to asymptomatic, SMR/SMR ratio = 3.9, 95%CI = 1.3–11), Hirschsprung disease (odds ratio (OR) 7.2, 95%CI = 1.04–55), pneumonia in the first year of life or recurrently in adulthood (OR = 7.6/19, 95%CI = 1.3–43/2.6–140) and autoimmunity in adulthood (OR = 39, 95%CI = 3.5–430). In conclusion, patients with CHH may develop adult-onset immunodeficiency or malignancy without preceding clinical symptoms of immune defect, warranting careful follow-up. Variable disease course and risk factors for mortality should be acknowledged.

## Introduction

Cartilage-hair hypoplasia (CHH) is a rare autosomal recessive chondrodysplasia, with combined immunodeficiency (CID), short stature, hair hypoplasia, anemia, increased risk of malignancies, and Hirschsprung disease. CHH is caused by mutations in *RMRP*, the gene encoding the RNA component of mitochondrial RNA-processing endoribonuclease (1). Disease prevalence is exceptionally high among the Amish and Finnish populations (2). The pathogenesis involves defective cell proliferation, impaired telomere machinery and abnormal gene regulation (3–5). Increased mortality in CHH relates to infections in childhood and malignancies in young adulthood (6). Non-Hodgkin lymphoma and basal cell carcinoma (BCC) are the most common associated cancer types (7). In addition, lung disease related to bronchiectasis is an important contributor to morbidity (8).

Clinical and laboratory manifestations of CHH are highly variable, even among siblings with the same genotype (9). Immunodeficiency can range from asymptomatic to severe and prediction of clinical course remains a challenge. Some reports suggest Hirschsprung disease, short birth length or autoimmunity to influence disease severity (9–13). Studies exploring correlations between laboratory parameters and clinical phenotype provide contradictory results (12, 14–16).

While early hematopoietic stem cell transplantation (HSCT) is life-saving in severe CID, indications for HSCT in patients with milder immunodeficiency are less clear, and prospective studies with long-term follow-up are required to identify prognostic factors. We have conducted a prospective clinical and observational study in a large cohort of Finnish patients with CHH in order to describe clinical course and long-term outcome and to identify risk factors for early mortality.

## Materials and Methods

### Study Cohort and Research Setting

All subjects and/or caregivers gave written informed consent in accordance with the Declaration of Helsinki. The protocol was approved by the Institutional Ethics Committee.

**Figure 1:**
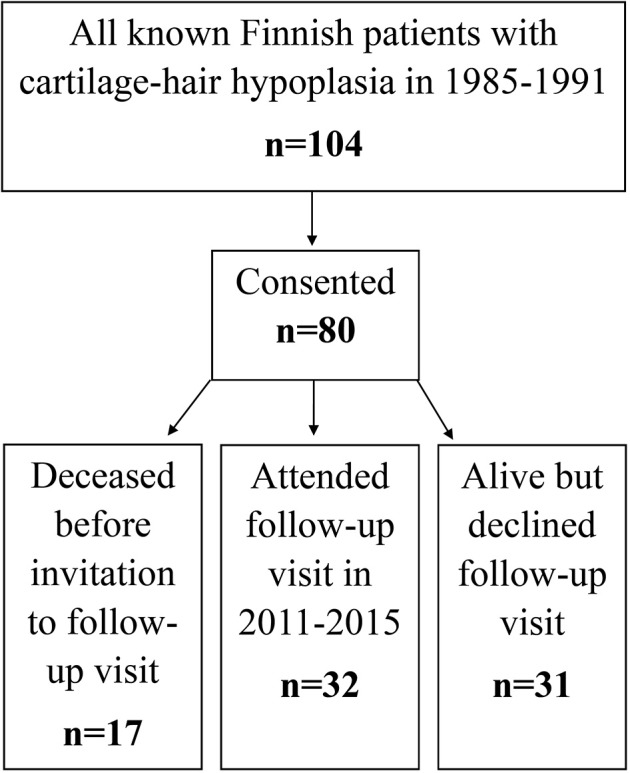
Flowchart of patient recruitment and data sources used in the study.

This prospective cohort study recruited in 1985–1991 all Finnish patients with CHH (*n* = 104) based on a nation-wide study (Figure 1) (2). Those who agreed to participate (*n* = 80) were included and underwent in 1985–1991 interview, clinical examination and blood sampling; *RMRP* mutations were confirmed by Sanger sequencing when the gene was discovered in 2001 (1).

In 2011–2015 we invited all surviving patients, 63 of the 80 patients, to follow-up visits. Those who attended (*n* = 32) underwent structured interview, clinical examination and blood sampling. For the remaining 31 patients, clinical and laboratory data were collected from hospital records (Figure 1).

The cohort of 24 CHH patients who were identified in 1985–1991 but did not agree to participate at baseline were followed for mortality based on register data only.

Parts of the clinical and laboratory data from the baseline and follow-up visits have been published previously in cross-sectional study settings (9, 13, 17); in this study all available data from the study visits were combined with data from normal clinical follow-up visits at any health care provider and with data collected from various registries (see below).

### Register-Based Data Collection

For all 80 patients, we obtained health information from two Finnish National Medical Databases. The Finnish National Care Register for Health Care has since 1969 recorded activities of health centers, hospitals, and other institutions providing inpatient and home-nursing care in the whole country. Outpatient primary health care data were derived from the Finnish National Register of Primary Health Care Visits, which covers all health centers in Finland since 2011. Database information included health service providers, dates of visits, diagnoses, as well as diagnostic and therapeutic procedures, focusing on conditions associated with immunodeficiency. We then collected all patients' health records from all identified hospitals for more detailed analysis. The primary outcome was death relating to immunodeficiency, combining deaths from infections, malignancy, and lung disease. Other recorded and analyzed features were the development of lymphoma, the development of skin cancer, other malignancies besides lymphoma and skin cancer, all types of infections, respiratory, gastrointestinal and autoimmune diseases, anemia, diagnostic and surgical procedures, and therapy for immunodeficiency.

Additionally, data on mortality were obtained from the Statistics Finland, covering the period from 1971. Data from all registries were available to the end of 2016.

### Clinical Categorization

Throughout the study, we used the term “children” for individuals aged <18 years and “adult” after 18th birthday. We and others have previously demonstrated that laboratory immunologic parameters are highly variable in patients with CHH and correlate poorly with the severity of clinical manifestations (12, 14, 17). In addition, categorization of patients based on solely clinical symptoms has the advantage of being easily applicable in clinical work. We therefore classified study subjects into three groups according to clinical symptoms of immunodeficiency without taking into account the results of laboratory tests: (1) clinically asymptomatic immunodeficiency (defined as no increased incidence of infections and further referred to as “*asymptomatic immunodeficiency*”), (2) clinical humoral immunodeficiency (recurrent respiratory tract infections and/or sepsis, further referred to as “*humoral immunodeficiency*”), and (3) clinical CID (additional features of autoimmunity or opportunistic infections, further referred to as “*CID*”). Opportunistic infections included thrush beyond the first 6 months of life, Candida esophagitis, refractory warts, recurrent mucocutaneous herpes simplex virus infections, as well as severe varicella. Recurrent pneumonia was defined as ≥2 episodes within a year or as ≥3 episodes ever. Recurrent otitis media and/or rhinosinusitis were defined as ≥3 episodes within 6 months, ≥4 within a year or ≥10 ever. Refractory warts were defined as warts persisting for years and requiring multiple treatment courses.

This clinical immunodeficiency classification was applied (1) at recruitment (for Kaplan–Meier analysis), (2) at 18 years (to evaluate symptoms in childhood), (3) after 18 years (to evaluate symptoms in adulthood) and (4) at the end of the follow-up (to evaluate symptoms during lifetime).

### Laboratory Data

Blood samples included complete blood counts (flow cytometry), and immunoglobulin levels (nephelometry) and lymphocyte subsets (flow cytometry), as described previously (17). We also searched hospital records for additional results of such tests, as well as of lymphocyte proliferation studies and vaccine responses. We excluded children <4 years of age from the analysis of immunoglobulin G subclasses and subjects with ongoing immunoglobulin substitution from the analysis of immunoglobulins, subclasses and vaccine responses.

### Statistical Methods

Calculation of person-years of follow-up for each patient was started from the date of the first research visit or 1 January 1987 (whichever was first) and ended at date of death or on 31 December 2016 (whichever was earlier). Observed numbers of deaths and person-years were stratified by sex, 5-year age groups and 5-year calendar periods. The expected numbers of deaths (overall and specific causes) for each stratum were calculated by multiplying person-years by the corresponding sex, age and period specific mortality rates in the general population produced by the Statistics Finland. The standardized mortality ratios (SMRs) were calculated as an observed to expected ratio. We stratified the causes of death into 54 categories of the longitudinal time series of Statistics Finland (18).

Exact 95% confidence intervals (95%CI) for the SMRs were defined with the assumption that the number of observed deaths followed a Poisson distribution.

To test the significance of difference between SMRs in subgroups of patients, we calculated SMR/SMR ratios and their 95%CIs.

We then analyzed all relevant and available clinical and laboratory features, categorized as (1) present in the first year of life, (2) present in childhood, (3) present in adulthood and (4) present at any age during lifetime/follow-up time, for their association with the immunodeficiency-related mortality. The analyses were adjusted for the patients' sex and age at the time of outcome.

Variables analyzed in the univariate models included birth length and most recent height, all types of infections, gastrointestinal, respiratory and autoimmune diseases, and all laboratory immunologic parameters. The univariate model analyzed each variable separately and those found to be significant were analyzed for correlations. In the pairs of significantly (correlation coefficient ≥0.6) correlating variables, we excluded those with lower OR. The remaining variables were then included in the multivariate model of the regression analysis. The multivariate analyses were performed separately for clinical features in childhood and in adulthood.

Statistical analyses were performed with IBM SPSS version 23 software.

## Results

### Characteristics of the Study Cohort

The study cohort consisted of 80 patients (35 males, 45 females) with CHH, all ethnic Finns. Median age at baseline recruitment was 14.6 years (range from 2 weeks to 49.6 years). Median duration of follow-up was 27.5 years (range 0.2–31.0 years) for all subjects and 29.2 years (range 25.6–31.0 years) for the surviving patients. All patients carried the n.71A>G *RMRP* variant, either in homozygosity (*n* = 62, 78%) or compounded with n.263G>T (*n* = 16, 20%) or a duplication at−13 (TACTCTGTGA) (*n* = 2, 2%) (NCBI reference sequence: NR_003051.3).

### Clinical Course

Clinical and laboratory features of the study cohort were consistent with several previous reports on Finnish patients with CHH (8, 9, 14, 15, 17) and are therefore not further outlined or discussed here in detail.

**Figure 2:**
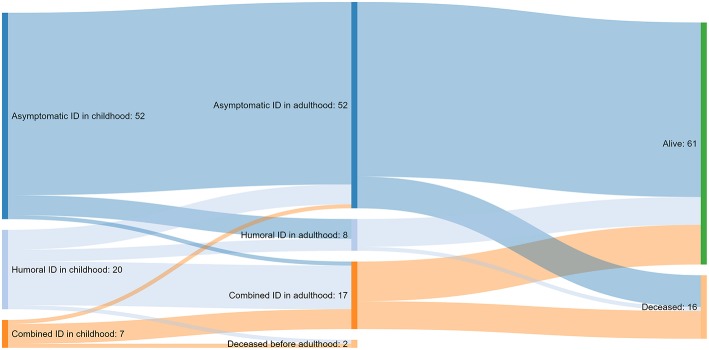
A Sankey diagram illustrating the course of immunodeficiency (ID) from childhood to adulthood and the outcome at the end of the follow-up for 79 of the 80 patients with cartilage-hair hypoplasia; data not available for one patient.

In a significant proportion of patients (17/79, 22%), clinical features of immunodeficiency progressed over time (Figure 2). Data on childhood symptoms were incomplete for one patient, who was therefore excluded from the analysis of clinical course.

More than half of the patients (46/80, 57%) had asymptomatic immunodeficiency by the end of the follow-up. Fifteen (19%) and 19 (24%) patients presented with humoral immunodeficiency and CID, respectively.

Of the 52 subjects with asymptomatic immunodeficiency in childhood, only 34 (65%) were alive, cancer-free and had no symptoms of immunodeficiency at the end of follow-up. Of the remaining 18 patients, 15 have been diagnosed with cancer, and of them, four deceased from cancer. Another four of these 18 patients died of immunodeficiency-unrelated causes like accidents (*n* = 2), circulatory system disease (*n* = 1) and alcohol-related disease (*n* = 1). Five of the surviving 10 patients from this group have developed symptoms of humoral immunodeficiency.

More than half of the patients with humoral immunodeficiency in childhood progressed to CID in adulthood. Most patients with CID in childhood continued to suffer from CID in adulthood associated with poor prognosis (4/7 developed cancer and 4/7 died).

Only a few patients had been treated with prophylactic antibiotics (*n* = 2), immunoglobulin replacement therapy (*n* = 3) or both (*n* = 4). Median time from the onset of recurrent respiratory tract infections to the commencement of treatment was 8 years for prophylactic antibiotics (range 0.5–23 years) and 16 years for immunoglobulin replacement therapy (range 5–65 years). None of the four patients with bronchiectasis in this group had received treatment prior to the diagnosis of bronchiectasis. None of the study patients underwent HSCT.

### Malignancy

Malignancy was diagnosed in 21 out of 80 patients (31%), all in adulthood. Of the 15 individuals with non-skin cancer, eight had no preceding clinical symptoms of immunodeficiency. Nine patients (11%) developed lymphoma: seven were non-Hodgkin and five were fatal. Median age at lymphoma diagnosis was 32.5 years (range 20.2–45.4 years). In all surviving lymphoma patients, the diagnosis was made either during a scheduled screening or after evaluation for non-specific mild symptoms.

Skin cancer was diagnosed in 15 out of 80 patients (19%), with 11 subjects presenting with BCC, two with squamous cell carcinoma and another two with both. All skin cancers were located either on the face, head, or upper limbs.

Other malignancies included single cases of lip squamous cell carcinoma, myelodysplasia, neuroendocrine carcinoma, plasmacytoma, thyroid carcinoma, and vocal cord carcinoma.

Altogether, five patients developed both skin and non-skin malignancies, at variable timelines and in random order.

### Mortality

**Figure 3:**
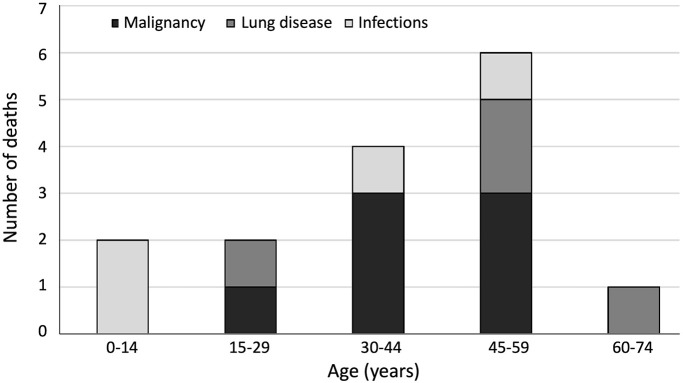
Age distribution of immunodeficiency-related causes of death (*n* = 15) in 80 patients with cartilage-hair hypoplasia.

Altogether, 20 of the 80 patients deceased before the end of 2016. According to the analysis of hospital records, immunodeficiency-related causes of death included pneumonia in children (*n* = 2, at 2.3 and 14.5 years), and malignancy (*n* = 7), respiratory disease (*n* = 4) and pneumonia (*n* = 2) in adults (Figure 3). The four patients who died from lung disease had been diagnosed with lung emphysema (*n* = 2) and bronchiectasis (*n* = 4). Deaths from malignancy were due to lymphoma (*n* = 5), neuroendocrine carcinoma (*n* = 1) and lip squamous cell carcinoma (*n* = 1). Median age at death from immunodeficiency-related causes was 40.9 years, being 24.4 years for infections, 40.9 years for malignancies and 52.8 years for lung disease. In addition, in five adults, the cause of death was accidental and/or unrelated to CHH.

Compared with the general population, patients with CHH had significantly higher mortality rates (SMR = 7.0, 95%CI = 4.3–11), especially due to lymphoid neoplasms (SMR = 60, 95%CI = 16–150) and diseases of the respiratory system (SMR = 46, 95%CI = 9.5–130) (Table 1). The overall SMR was similarly increased in males (SMR = 7.1, 95%CI = 3.5–13) and females (SMR = 7.0, 95%CI = 3.2–13).

**Table 1:** Mortality in a cohort of 80 patients with cartilage-hair hypoplasia in 1987–2016 (2,048 person-years)^a^.

**Cause of death**	**Obs**	**Exp**	**SMR**	**95%CI**
All causes	20	2.85	7.0	4.3–11^***^
Age 0–14	2	0.09	21	2.6–77^**^
Age 15–29	4	0.48	8.3	2.3–21^**^
Age 30–44	6	0.79	7.6	2.8–17^***^
Age 45–74	8	1.48	65.4	2.3–11^***^
All diseases	17	1.93	8.8	5.1–14^***^
Infections	0	0.02	0.0	0.0–160
Neoplasms	7	0.69	10	4.1–21^***^
Malignant neoplasms	6	0.68	8.8	3.2–19^***^
Lymphoid/hematopoietic neoplasms	4	0.07	60	16–150^***^
Malignant neoplasms of lip	1	0.01	75	1.9–420^*^
Malignant melanoma of skin	0	0.02	0.0	0.0–230
Malignant neoplasm of breast	0	0.12	0.0	0.0–31
Other malignant neoplasms	1	0.13	7.7	0.2–43
Other neoplasms	1	0.01	98	2.5–540^*^
Endocrine, nutritional, and metabolic diseases	0	0.06	0.0	0.0–65
Circulatory system diseases	2	0.47	4.3	0.5–16
Respiratory system diseases	3	0.07	46	9.5–130^***^
Diseases of the digestive system	0	0.05	0.0	0.0–78
Congenital malformations	4	0.05	76	21–190^***^
Alcohol–related diseases	1	0.33	3.0	0.1–17
Accidents and violence	3	0.9	3.3	0.7–9.8

*95%CI 95% confidence interval; Exp, expected number of deaths; Obs, observed number of deaths; SMR, standardized mortality ratio*.

a *For four patients the registered underlying cause of death was “congenital malformations,” referring to CHH, which was used in the SMR analysis. However, according to the patients' hospital records the cause of death was pneumonia (n = 3) and lung disease (n = 1). For another patient, the registered cause of death was “diseases of circulatory system” but hospital records indicated that patient had deceased from pneumonia*.

* *p < 0.05*,

** *p < 0.01*,

*** *p < 0.001*.

To exclude selection bias, we analyzed separately mortality rates for the 24 Finnish patients with CHH who did not participate the baseline study in 1985–1991. Eight of them deceased during follow-up (8/24, 33%). Causes of death included malignancy (*n* = 3), accidents (*n* = 2), alcohol-related diseases (*n* = 1), circulatory diseases (*n* = 1), and congenital malformations (*n* = 1).

### Factors Associating With Adverse Outcomes

Risk factors for immunodeficiency-related death included Hirschsprung disease and pneumonia in the first year of life or recurrently in adulthood, as well as autoimmunity in adulthood (Tables 2, 3). In addition, all-cause mortality was significantly higher in those with severe short stature at birth (<-4.0 standard deviation, adjusted for gestational age) and in patients with CID (Table 4).

**Table 2:** Risk factors for immunodeficiency-related death in a cohort of 80 patients with cartilage-hair hypoplasia.

**Risk factors for immunodeficiency-related death**	**OR**	**95%CI**	**Spearman's correlation coefficient**		**Included in multivariate analysis**
*Clinical features in the first year of life*			0.4: A/B	**0.6: B/C**	
A. Hirschsprung disease	15	1.8–130^*^		0.4: B/D, B/F, D/K	+
B. Pneumonia	7.8	1.7–36^**^		0.3: A/F, D/J	+
*Clinical features in childhood*				0.2: A/D, B/I, B/J	
C. Pneumonia	8.2	2.3–29^**^	**0.7: C/E**	0.1: A/I, A/J, B/K, D/F, D/I	−
D. Recurrent rhinosinusitis	6.8	1.1–43^*^	0.3: C/D	0.0: AK	+
E. CID	6.7	1.2–38^*^			−
*Clinical features in adulthood*					
F. Autoimmune disease	42	4.2–410^**^	**0.6: F/G, F/L, H/I**		+
G. CID	6.8	1.8–26^**^	0.4: J/K, I/J, I/K		−
H. Pneumonia	5.9	1.6–22^**^	0.3: F/I		−
I. Recurrent pneumonia	22	3.3–140^**^	0.1: F/J, F/K		+
J. Recurrent otitis media	11	1.8–63^*^			+
K. Recurrent rhinosinusitis	5.5	1.4–22^*^			+
L. Low serum levels of IgG	23	2.3–230^**^			−

*All study variables were first analyzed by univariate analysis only adjusted for age and gender. Variables proven significant were then grouped by the time of onset. Significantly (correlation coefficient >0.6, marked in bold) correlating variables were excluded and the remaining variables were combined for the multivariate analysis. 95%CI, 95% confidence interval; CID, combined immunodeficiency; Ig, immunoglobulin; OR, odds ratio*.

* p < 0.05,

** *p < 0.01*.

**Table 3:** Risk factors for immunodeficiency-related death in a cohort of 80 patients with cartilage-hair hypoplasia, analyzed separately in childhood and adulthood.

**Risk factors for immunodeficiency-related death (*n* = 15)**	**Prevalence in patients with outcome, N (%)**	**Prevalence in patients without outcome, N (%)**	**OR**	**95%CI**
*Clinical features in childhood^a^*				
Hirschsprung disease	4/15 (27)	2/65 (3)	7.2	1.04–55^*^
Pneumonia in the first year of life	5/14 (36)	4/63 (6)	7.6	1.3–43^*^
Recurrent rhinosinusitis	4/14 (29)	3/61 (5)	1.6	0.1–21
*Clinical features in adulthood^b^*				
Autoimmune disease	5/13 (39)	1/65 (2)	39	3.5–430^**^
Recurrent pneumonia	5/13 (39)	2/65 (3)	19	2.6–140^**^
Recurrent otitis media	4/13 (31)	3/65 (5)	4.2	0.4–43
Recurrent rhinosinusitis	6/13 (46)	9/64 (14)	2.4	0.3–18

*CI, confidence interval; Ig, immunoglobulin; N, number; OR, odds ratio*.

* *p < 0.05*,

** p < 0.01.

a *Model included age, gender, and on/off variables for Hirschsprung disease, pneumonia in the first year of life, and recurrent rhinosinusitis*.

b *Model included age, gender, and on/off variables for autoimmune disease, recurrent pneumonia, recurrent otitis media, and recurrent rhinosinusitis*.

**Table 4:** Mortality from selected causes in 80 patients with cartilage-hair hypoplasia categorized by birth length standard deviation score and by the degree of immunodeficiency at recruitment.

	**Obs**	**Exp**	**SMR**	**95%CI**	**SMR/SMR ratio (95%CI)**
**CAUSES OF DEATH IN VARIOUS BIRTH LENGTH CATEGORIES ^a^**					
**Normal birth length (*****n*** **= 28)**					Normal vs. moderately short birth length: 1.8 (0.5–6.2).
All deaths	6	1.47	4.1	1.5–8.9^**^	
Neoplasms	3	0.44	6.7	1.4–20^*^	
Lymphoid/hematopoietic neoplasms	2	0.04	53	6.4–190^**^	
Respiratory diseases	1	0.04	24	0.6–130	
**Moderately short birth length (*****n*** **= 39)**					
All deaths	8	1.11	7.2	3.1–14^***^	
Neoplasms	3	0.20	15	3.1–44^**^	
Lymphoid/hematopoietic neoplasms	2	0.02	87	11–320^***^	
Respiratory diseases	0	0.02	0.0	0.0–190	
**Severely short birth length (*****n*** **= 13)**					Normal vs. severely short birth length: 5.4 (1.5–20).
All deaths	6	0.27	22	8.1–48^***^	
Neoplasms	1	0.05	20	0.5–110	
Lymphoid/hematopoietic neoplasms	0	0.01	0	0.0–600	
Respiratory diseases	2	0.00	430	52–1600^***^	
**CAUSES OF DEATH IN VARIOUS CATEGORIES OF IMMUNODEFICIENCY AT RECRUITMENT**					
**Asymptomatic immunodeficiency (*****n*** **= 52)**					Asymptomatic vs. humoral immunodeficiency: 1.2 (0.1–5.7).
All deaths	10	2.07	4.8	2.3–8.9^***^	
Neoplasms	3	0.49	6.2	1.3–18^*^	
Lymphoid/hematopoietic neoplasms	2	0.05	43	5.2–150^**^	
Respiratory diseases	2	0.05	44	5.3–160^**^	
**Symptoms of humoral immunodeficiency (n = 18)**					
All deaths	2	0.34	5.9	0.7–21	
Neoplasms	0	0.09	0	0.0–41	
Lymphoid/hematopoietic neoplasms	0	0.01	0	0.0–400	
Respiratory diseases	0	0.01	0	0.0–540	
**Symptoms of combined immunodeficiency (n = 10)**					
All deaths	8	0.43	19	8.0–36^***^	Asymptomatic vs. .combined immunodeficiency: 3.9 (1.3-11).
Neoplasms	4	0.12	34	9.3–87^***^	
Lymphoid/hematopoietic neoplasms	2	0.01	180	22–650^***^	
Respiratory diseases	1	0.01	79	2.0–440^*^	

*95%CI 95% confidence interval, Exp, expected number of deaths; n, number of patients; Obs, observed number of deaths; SMR, standardized mortality ratio*.

a *Patients birth length was adjusted for gestational age and compared with normative data for the Finnish population (19) and the standard deviation (SD) score was applied for analysis. Patients were categorized as having normal (SD score higher than-2.0), moderately short (SD-2.0 to−4.0) or severely short (SD lower than-4.0) birth length*.

* *p < 0.05*,

** *p < 0.01*,

*** *p < 0.001*.

**Figure 4:**
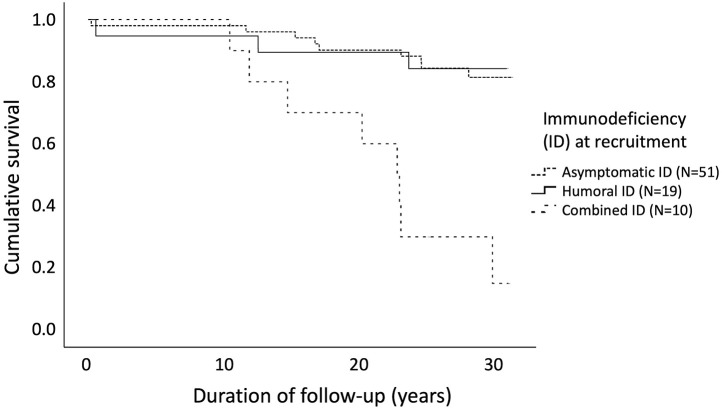
Cancer-free survival in 80 patients with cartilage-hair hypoplasia differed significantly [log rank test χ^2^(2) = 24.8, 95%CI = 18.4–26.9, *p* < 0.0001] depending on the severity of immunodeficiency at recruitment. Mean age at recruitment was 17.3 (range 0.0–42.3), 13.3 (range 1.0–46.0), and 22.4 (range 4.1–49.6) years for patients with asymptomatic, humoral and combined immunodeficiency, respectively.

We performed a Kaplan-Meier analysis (log rank test) of 80 patients, categorizing them by the degree of immunodeficiency (asymptomatic, humoral or CID) assessed at the day of recruitment (Figure 4). The age distribution at recruitment was similar across these categories of immunodeficiency, as evaluated by Kruskal–Wallis test (*p* = 0.159). Mean age at recruitment was 17.3 (range 0.0–42.3), 13.3 (range 1.0–46.0), and 22.4 (range 4.1–49.6) years for patients with asymptomatic, humoral and combined immunodeficiency, respectively. Survival rates for these three groups differed significantly [$\chi_{\left(2\right)}^{2}$ = 24.8, 95%CI = 18.4–26.9, *p* < 0.0001], asymptomatic children having the best and children with CID the worst outcome.

## Discussion

This prospective study describes the clinical course and outcomes of 80 Finnish patients with CHH followed for 30 years, and demonstrates high overall mortality, mostly from malignancies and lung disease. We also report several risk factors for mortality, providing clinicians with useful prognostic markers.

The degree of immunodeficiency in CHH is highly variable. All previously reported Finnish patients with CHH were either homozygous or heterozygous for the most prevalent *RMRP* mutation n.71A>G, suggesting phenotype variations to be independent of the genotype. Polymorphisms in *RMRP* gene and non-allelic modifiers can explain the variable clinical features in patients with identical *RMRP* variants (20, 21). Similar phenotype-genotype inconsistency has been reported in non-Finnish cohorts of patients with CHH, who demonstrate a much broader spectrum of *RMRP* mutations (22, 23).

Large proportion of patients in our cohort remained clinically asymptomatic for decades. However, some of them progressed to adult-onset immunodeficiency, while others developed fatal malignancy. This underscores the importance of careful follow-up of also those patients who do not exhibit any clinical symptoms of immunodeficiency. The initial humoral immunodeficiency in childhood often evolved into CID in adulthood and therefore should not provide a false sense of “mild” disease. Patients with CID in childhood had the most severe outcomes with high mortality and might benefit from early interventions including HSCT.

In accordance with earlier studies (7, 24), malignancy was common in our cohort. Survival from lymphoma (4/9, 44%) was better than previously reported (7, 24), and attributable to early diagnosis. This highlights the value of regular screening for lymphoma by abdominal ultrasound, which allowed for early diagnosis in two asymptomatic patients. It also emphasizes the need for an aggressive diagnostic approach in symptomatic patients, illustrated by the early detection of lymphoma by gastroscopy performed for vague abdominal pain. Lymphoma development has been reported in CHH children as young as six years (25), as well as in several older children (9, 26, 27). We therefore recommend that screening for lymphoma should begin at 5 years of age.

Many patients with no symptoms of immunodeficiency developed malignancy during follow-up, suggesting that the pathogenesis of lymphoma in CHH is multifactorial and that the severity of immunodeficiency correlates poorly with the risk of lymphoma. Lymphoproliferative disorders in patients with CHH can be Epstein-Barr virus-driven in some (27, 28), but not all cases (29). Therefore, not only impaired viral suppression, but other mechanisms, such as chromosomal instability, may play a role (30, 31). The impaired telomere biology has been reported in patients with CHH and may contribute to the increased risk of malignancies (4, 5).

None of the subjects in our cohort had received HSCT. However, for several patients, HSCT has been considered, unfortunately late in the disease course, and deemed impossible due to poor health status. The outcome of HSCT in patients with CHH has been reported in a series of 3, 6, 13 and 16 patients with survival rates of 100, 100, 83, and 63%, respectively, evaluated at 5–20 years, 1.5–22 years, 1.8–14 years and 1.6–16 years post-transplant, respectively (16, 26, 32, 33). The majority of surviving patients achieved full reconstitution of B and T cell numbers and function, the quality of life was improved, and no cancer cases have yet been reported. Early HSCT can be lifesaving in CHH, while HSCT performed after the onset of opportunistic infections can be fatal (34). However, given the mild clinical course of some patients with CHH and good survival after HSCT in adults with PID (35), a more conservative approach could be possible, with careful observation and timely detection of disease progression. Therefore, knowledge on the risk factors for the development of severe complications is crucial to detect subjects most likely to benefit from HSCT.

We recognize several limitations in our study. A significant part of health data was collected from registries and health records from other hospitals, and we cannot ensure the completeness of the obtained data. The number of patients with primary outcomes and the number of events in the subgroups of patients were small, which should be considered when interpreting the results of multivariate analyses.

The strengths of our study include the long follow-up, allowing for identification of risk factors for adverse outcomes. Data were derived from several sources, including patient interviews, causes-of death and health registries, as well as from all available hospital records. The use of Finnish National Health Registries allowed excellent data accuracy, coverage and completeness (36).

In conclusion, we describe variable patterns of disease course, highlighting the cases with adult-onset immunodeficiency or development of malignancy in otherwise clinically asymptomatic patients. We therefore recommend that patients with CHH who show no clinical symptoms of immunodeficiency should also be screened annually for malignancy including basic physical and laboratory evaluation (at least complete blood count and sedimentation rate) and imaging or biopsy as needed based on symptom history. In addition, we demonstrate for the first time that immunodeficiency-related lung disease is an important cause of mortality in adults with CHH. Hence, patients should undergo regular pulmonary evaluation, including diffusing capacity of the lungs for carbon monoxide and chest imaging as discussed previously (8). Our categorization of patients based on clinical features of immunodeficiency predicted overall survival and can thus be implicated in clinical practice. Most importantly, we provide clinicians data on risk factors for mortality; these can be used to discuss prognosis and to make management decisions.

## Data Availability

All datasets generated for this study are included in the manuscript and/or the supplementary files.

## Ethics Statement

This study was carried out in accordance with the recommendations of the Ethics Committee for gynecology and obstetrics, pediatrics and psychiatry at the Helsinki University Hospital and University of Helsinki with written informed consent from all subjects. All subjects gave written informed consent in accordance with the Declaration of Helsinki. The protocol was approved by the Ethics Committee for gynecology and obstetrics, pediatrics and psychiatry at the Helsinki University Hospital and University of Helsinki.

## Author Contributions

OM designed the study. SV collected and analyzed the data and drafted the manuscript. EP calculated standardized mortality ratios. SV, MT, PK, EP, and OM contributed to the writing of the manuscript and approved the final version.

### Conflict of Interest Statement

The authors declare that the research was conducted in the absence of any commercial or financial relationships that could be construed as a potential conflict of interest.

## Abbreviations

BCC: basal cell carcinoma
CHH: cartilage-hair hypoplasia
CI: confidence interval
CID: combined immunodeficiency
HSCT: hematopoietic stem cell transplantation
SMR: standardized mortality ratio.
